# The synthesis and application of (*E*)-*N*′-(benzo[*d*]dioxol-5-ylmethylene)-4-methyl-benzenesulfonohydrazide for the detection of carcinogenic lead†

**DOI:** 10.1039/c9ra09080k

**Published:** 2020-02-03

**Authors:** Mohammed M. Rahman, Mohammad Musarraf Hussain, Muhammad Nadeem Arshad, Abdullah M. Asiri

**Affiliations:** Chemistry Department, Faculty of Science, King Abdulaziz University P.O. Box 80203 Jeddah 21589 Saudi Arabia mmhussainkau@gmail.com; Center of Excellence for Advanced Materials Research, King Abdulaziz University P.O. Box 80203 Jeddah 21589 Saudi Arabia; Department of Pharmacy, Faculty of Life and Earth Sciences, Jagannath University Dhaka-1100 Bangladesh

## Abstract

In this study, noble ligands of (*E*)-*N*′-(benzo[*d*]dioxol-5-ylmethylene)-4-methyl-benzenesulfonohydrazide (BDMMBSH) were prepared *via* a simple condensation method using benzo-[*d*][1,3]-dioxole carbaldehyde, benzenesulfonylhydrazine (BSH), and 4-methyl-benzenesulphonylhydrazine (4-MBSH) in good yield, which were crystallized in acetone, EtOAc, and EtOH. The BDMMBSH derivatives were characterized using different spectroscopic techniques, such as ^1^H-NMR, ^13^C-NMR, FTIR, and UV-Vis spectroscopy, and their crystal structures were analyzed using the single crystal X-ray diffraction method (SCXRDM). Subsequently, the BDMMBSH compounds were used for the significant detection of the carcinogenic heavy metal ion, lead (Pb^2+^), *via* a reliable electrochemical approach. A sensitive and selective Pb^2+^ sensor was developed *via* the deposition of a thin layer of BDMMBSH on a GCE with the conducting polymer matrix Nafion (NF). The sensitivity, LOQ, and LOD of the proposed sensor towards Pb^2+^ were calculated from the calibration curves to be 2220.0 pA μM^−1^ cm^−2^, 320.0 mM, and 96.0 pM, respectively. The validation of the BDMMBSH/GCE/NF sensor probe was performed *via* the selective determination of Pb^2+^ in spiked natural samples with a satisfactory and rational outcome.

## Abstract 2

A sensitive cationic sensor was developed by BDMMBSH onto GCE with 5% Nafion using electrochemical method, which was validated with the selective determination of Pb^2+^ in spiked samples and found satisfactory results.

## Graphical abstract

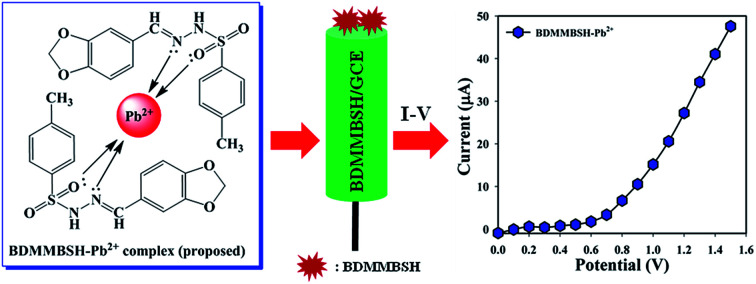

## 1. Introduction

Currently, toxic metal ions are a significant source of pollution in nature, which originate from impure water, foodstuffs, and soil.^1^ Among them, the presence of lead ions (Pb^2+^) is a key concern to human health owing to their extremely poisonous characteristics at low concentrations. Exposure to Pb^2+^ in humans (children) may cause different adverse effects and toxicity, for example brain issues, behavioral problems, cardiovascular problems, learning disability, muscle paralysis, memory failures, irritability, reproductive issues, and hearing disorders.^2,3^ Pb^2+^ can accumulate in the kidneys and bones, and can lead to damage of the CNS and renal activities. Traditional methodical procedures such as electrochemical techniques, GFAAS, ICP-MS, ICP-AES, ASV, and X-ray fluorescence spectrometry (XRFS) have been reported in previous studies for the detection of Pb^2+^. However, these methods require costly operation, complicated instrumentation, skilled manpower, and complicated sample preparation.^4,5^ Thus, it is necessary to design a new approach with good simplicity, sensitivity, and selectivity for the detection of Pb^2+^. The electrochemical approach (sensor) is an investigative tool that consists of finding elements such as biological, chemical, and metal ions and detector particles (*i.e.* transducers). A transducer can shift the response of the desired elements into a detectable signal that can be made from different materials (electrodes, nanoparticles, small organic molecules, and thin films). Electrochemical sensors represent a capable advancement to complement the previously published procedures owing to their easy instrumentation, low cost, high selectivity and sensitivity, and significant properties for the successful detection of heavy metal ions.^6–14^

In this research, a simple, sensitive, and selective Pb^2+^ sensor was developed based on (*E*)-*N*′-(benzo[*d*]dioxol-5-ylmethylene)-4-methyl-benzenesulfonohydrazide derivatives modified on a GCE with 5% conducting Nafion polymer matrix *via* an electrochemical approach under ambient conditions.

## 2. Experimental

### 2.1 Materials and methods

Chemicals including benzo-[*d*][1,3]-dioxole carbaldehyde, BSH, 4-MBSH, AgNO_3_, AuCl_3_, CaCl_2_, FeCl_3_, MgCl_2_, Pb(NO_3_)_2_, ZnSO_4_, SnCl_2_, EtOH, NaH_2_PO_4_, Na_2_HPO_4_, and NF (5.0 weight% in water and lower aliphatic alcohol containing 45.0% water) were purchased from Sigma Aldrich Company, KSA and used as received. A mother solution of toxic metal ions (100.0 mM and 10.0 mL) was made from the respective purchased chemical with distilled water, DSTW (10.0 mL). ^1^H-NMR and ^13^C-NMR spectra were recorded at 300 K on an ASCEND NMR machine (400 and 850 MHz, respectively) and chemical shifts reported in ppm with a common solvent signal as a reference. FTIR and UV-Vis experiments were conducted using a Thermo Scientific NICOLET iS50 and Evolution 300 UV-Vis spectrophotometer. Electrochemical (*I*–*V*) examination was performed to detect Pb^2+^ at a selective point with a BDMMBSH/GCE/NF sensor using a Keithley electrometer.

### 2.2 Crystallography analysis

A suitable sample of two of the newly synthesized and crystallized (*E*)-*N*′-(benzo[*d*]dioxol-5-ylmethylene)-4-methyl-benzenesulfonohydrazide molecules was placed under a microscope and observed prudently to find good crystals. An appropriate crystal was selected and attached over fiber glass needles merged with a hollow copper tube having a magnetic base. This sample holder was mounted on an Agilent Super Nova diffractometer equipped with micro-focus Cu–Mo Kα radiation and data collection was accomplished using the CrysAlisPro software.^15^ Figures of the as-synthesized derivatives (4 and 5) were generated through PLATON, ORTEP inbuilt with WinGX and Olex^2^.^16–18^ C–H hydrogen atoms were positioned geometrically with C–H = 0.93, 0.97, and 0.96 Å and treated as riding atoms with Uiso (H) = 1.2 and 1.5 Ueq. for aromatic, methylene, and methyl carbon atoms, respectively. N–H hydrogen atoms were located through Fourier map and refined with N–H = 0.80 (2)–0.86 (2) Å with Uiso (H) = 1.2 Ueq. for nitrogen atoms. The crystal data were deposited in the Cambridge Crystallographic Data Centre with CCDC numbers 1589736 and 1589737 for 4 and 5, respectively.

### 2.3 Preparation of BDMMBSH derivative

#### 2.3.1 (*E*)-*N*′-(benzo[*d*]dioxol-5-ylmethylene)-benzenesulfonohydrazide (BDMBSH, 4)

A mixture of benzo[*d*][1,3]dioxole-5-carbaldehyde (526.9 mg, 3.51 mmol and 1.17 equiv.) and BSH (516.0 mg, 3.00 mmol, and 1.0 equiv.) was added to EtOH (30.0 mL) and continued stirring at R.T. for 2.5 h. A white precipitate was observed, and the reaction flask was left to stand to allow the formed precipitate to settle. The solvent was removed and cold MeOH (20.0 mL) was added with precipitate, and kept at open air to evaporate the solvent slowly. The obtained product was crystallized from MeOH and the obtained light-yellow powder was re-crystallized from acetone and EtOAc (50 : 50) to give the title compound 4 as a golden crystal (618.5 mg, 64%). EF = C_14_H_12_N_2_O_4_S, MW: 304.32 g mol^−1^, EA = C-55.25, H-3.97, N-9.21, O-21.03, S-10.54. ^1^H-NMR (400 MHz, DMSO-*d*_6_) *δ*: 11.16 (s, 1H), 7.94–7.82 (m, 3H), 7.69–7.57 (m, 3H), 7.11 (d, *J* = 1.6 Hz, 1H), 7.03 (dd, *J* = 8.1, 1.7 Hz, 1H), 6.92 (d, *J* = 8.0 Hz, 1H), 6.05 (s, 2H). ^13^C-NMR (101 MHz, DMSO-*d*_6_) *δ*: 191.45, 149.54, 148.34, 147.60, 139.43, 133.48, 129.66, 128.46, 127.68, 123.51, 108.85, 106.79, 105.34, 101.99. FTIR (neat) *ν*_max_ = 3200, 2904, 1695, 1600, 1497, 1435, 1315, 1285, 1198, 1100, 1025, 945, 802, 725, 690, 600, 485. UV-Visible (DMSO), *λ*_max_ = 316.0 nm.

#### 2.3.2. (*E*)-*N*′-(benzo[*d*]dioxol-5-ylmethylene)-4-methyl-benzenesulfonohydrazide (BDMMBSH, 5)

EtOH (25.0 mL) was added to a reaction mixture of benzo[*d*][1,3]dioxole-5-carbaldehyde (509.7 mg, 3.40 mmol, and 1.26 equiv.) and 4-MBSH (25.0 mL, 500.2 mg, 2.69 mmol, and 1.0 equiv.) and stirred at R.T. for 2.5 h. Then, the mixture was filtered and the solution was left in open air to let the solvent evaporate slowly. The obtained product was crystallized from EtOH to give the desired molecule 5 as a red crystal (664.0 mg, 74.0%). EF = C_15_H_14_N_2_O_4_S, MW = 318.35, EA = C-56.59, H-4.43, N-8.80, O-20.10, S-10.07.^1^H-NMR (400 MHz, DMSO-*d*_6_) *δ*: 11.26 (s, 1H), 7.83 (s, 1H), 7.80–7.75 (m, 2H), 7.41 (d, *J* = 8.1 Hz, 2H), 7.11 (d, *J* = 1.6 Hz, 1H), 7.03 (dd, *J* = 8.0, 1.6 Hz, 1H), 6.18 (s, 1H), 6.06 (s, 2H), 2.27 (s, 3H). ^13^C-NMR (101 MHz, DMSO-*d*_6_) *δ*: 190.92, 149.00, 146.85, 143.36, 136.11, 129.58, 128.04, 127.23, 122.93, 108.34, 106.29, 104.84, 102.29, 101.49, 20.95. FTIR (neat) *ν*_max_ = 3600, 3200, 2900, 1690, 1600, 1500, 1465, 1355, 1285, 1200, 1095, 1010, 910, 800, 675, 600, 445. UV-Visible (DMSO), *λ*_max_ = 314.0 nm.

### 2.4 Preparation and modification of GCE with BDMMBSH molecules

A series of phosphate buffer (PB) from slightly acidic to basic pH = 5.7, 6.5, 7.0, 7.5, and 8.0 was prepared from NaH_2_PO_4_, Na_2_HPO_4,_ and DSTW and the amount of PB = 10.0 mL was kept constant throughout the experiment. Primarily, a GCE was cleaned systematically with DSTW and acetone, and subsequently left in open air to dry (1.0 h). The dried GCE was deposited with a slurry (EtOH + BDMMBSH) and left again in open air (1.5 h) to dry. NF was added dropwise to the dried deposited electrode and it again was left in open air (2.0 h) for harmonized film development with comprehensive drying. The custom-made GCE and Pt wire were used as the working and counter electrode, respectively, to investigate the electrochemical (*I*–*V*) responses upon TMI exposure. Organic functional compounds have attracted significant attention due to their chemical, structural, electrochemical, and optical properties in terms of the attachment of active functional groups. Sensitivity and detection ability are directly dependent on the functional anchoring groups attached into the main frame carbon skeleton, which are prepared by reactant precursors under ambient conditions. BDMMBSH and BDMBSH were synthesized *via* a simple condensation method from benzo-[*d*][1,3]-dioxole carbaldehyde, benzenesulfonylhydrazine (BSH) and 4-methyl-benzenesulphonylhydrazine (4-MBSH) in good yield, which were crystallized in acetone, EtOAc, and EtOH systematically. The optical and functional properties of BDMMBSH and BDMBSH compounds have a huge significance as electrochemical sensor probes for selectivity to heavy metal ions and are superior to other ligands. The functional groups in BDMMBSH on its main carbon strands are responsible for enhancing the capture of heavy metal ions for the selective determination of Pb^2+^ cations.

## 3. Results and discussion

### 3.1 Studies of BDMMBSH derivatives

**Scheme 1:**
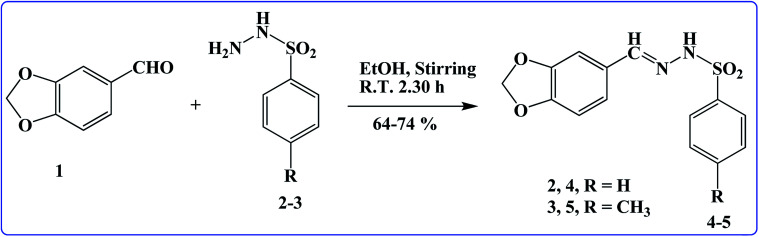
Synthesis of BDMMBSH molecules.

Two new title ligands (4 and 5) were prepared from benzo[*d*][1,3]dioxole-5-carbaldehyde (1), BSH (2), and 4-MBSH (3) in good yield *via* a simple route (Scheme 1).^19^ Using different spectroscopic procedures, molecules 4 and 5 were characterized, and their structures were confirmed finally *via* SCXRDM. The purity of the derivatives was confirmed from their spectra, which helped us to classify the existing protons in the molecules using the chemical coupling constant (*J*) and shift (*δ*). The N–H proton of the marked molecules 4 and 5 exhibited a singlet at *δ* 11.16 and 11.26, respectively. The aromatic protons in benzo[*d*][1,3]dioxole and phenyl groups of the as-prepared molecules (4 and 5) exhibited different signals (*δ*: 7.94–6.05 and 7.83–6.06) for 4 and 5, respectively. One more singlet was observed at *δ* 2.27, corresponding to three protons, which may be due to CH_3_ in the preferred molecule 5. ^13^C-NMR was also performed and the carbon atoms in the aromatic region were observed (Fig. S1–S4†). FTIR was also performed in the wavenumber range 4000–400 cm^−1^ to confirm the presence of the functional groups, which presented different bending and stretching peaks in the spectra. UV-Visible was conducted in DMSO in the wavelength range of 200–800 nm and *λ*_max_ was found to be 316.0 and 314.0 nm (Fig. S5 and S6†), respectively.

### 3.2 Analysis of crystal structure

**Fig. 1:**
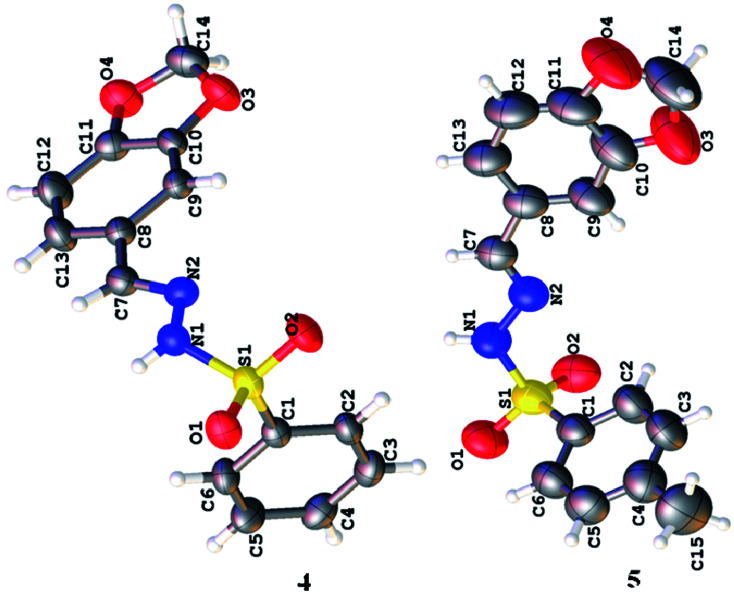
Molecular structures of the as-synthesized compounds.

**Fig. 2:**
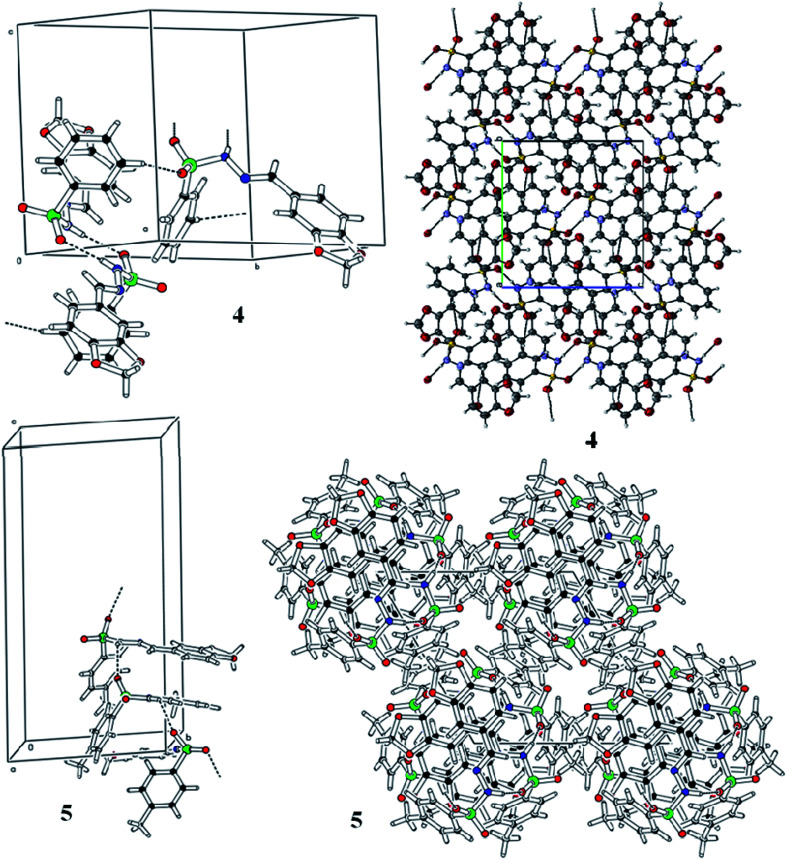
Hydrogen bonding pattern of the as-prepared molecules.

Spectroscopic analysis of the as-synthesized molecules (4 and 5) was performed *via* SCXRDM with the aim to determine the van der Waals interactions and geometry of the compounds in their unit cells. The structures of 4 and 5 were determined to be in a monoclinic and hexagonal crystal system with the space group *P*2_1_/c and *P*6_5_, respectively (Table 1). Both molecules belong to a homologous series since there is a difference in –CH_2_– between their molecular formulas. In general, the O–S–O angle in the sulfonamide family forces the other angles to adopt a tetrahedral geometry.^20–23^ The <O1–S1–O2 angles around the S atoms are 119.07 (8)° and 119.41 (19)° for molecules 4 and 5, respectively. The dihedral angle between the aromatic rings and 1,3-benzodioxol system are 72.46 (4)° and 76.26 (1)° (Fig. 1 and Table S1 and S2†). The root mean square deviation values for the fitted atoms of the dioxol ring are 0.0273 and 0.0369 Å for molecules 4 and 5, respectively. The two fused rings (benzo-dioxol) are twisted at 1.219 (7)° and 4.838 (6)° in 4 and 5, respectively. The molecules in the crystal structure of 4 connect each other *via* N–H⋯O and C–H⋯O interactions. The N–H⋯O interactions produce dimmers, which are further connected through C–H⋯O interactions very beautifully to produce a two-dimensional network along the *bc* plane. Only intermolecular hydrogen bonding was observed between the N–H of hydrazine and oxygen of the SO_2_ moiety in molecule 5. This linkage connects the molecules along the *c*-axis and forms an infinite chain in 5, and this arrangement produced ball-like shapes when viewed along the *ab* plane (Fig. 2 and Table 2).

**Table 1:** Crystal data and structure refinement of the as-synthesized molecules

Parameter	BDMBSH (4)	BDMMBSH (5)
ID	17075	17076
CCDC	1589736	1589737
EF	C_14_H_12_N_2_O_4_S	C_15_H_14_N_2_O_4_S
FW	304.32	318.34
Crystal system	Monoclinic	Hexagonal
Temperature/K	296(2)	296(2)
Space group	*P*2_1_/*c*	*P*6_5_
*a*/Å	10.3559(8)	11.4162(4)
*b*/Å	12.2616(9)	11.4162(4)
*c*/Å	10.7523(8)	20.9435(7)
Volume/Å^3^	1364.84(18)	2363.87(18)
*α*/°	90	90
*β*/°	91.528(7)	90
*γ*/°	90	120
*Z*	4	6
*ρ*_calc_ mg mm^−3^	1.481	1.342
Crystal size/mm^3^	0.48 × 0.29 × 0.20	0.40 × 0.31 × 0.24
*F*(000)	632.0	996.0
*μ*/mm^−1^	0.255	0.224
2*θ* range for data collection	6.458 to 58.3°	6.356 to 58.142°
Reflections collected	6171	5104
Index range	−8 ≤ *h* ≤ 14, −15 ≤ *k* ≤ 16, −14 ≤ *l* ≤ 14	−5 ≤ *h* ≤ 8, −10 ≤ *k* ≤ 11, −15 ≤ *l* ≤ 17
Independent reflections	3236 [*R*(int) = 0.0229]	3354 [*R*(int) = 0.0378]
Goodness-of-fit on *F*^2^	1.020	1.011
Data/restraints/parameters	3236/0/193	3354/1/201
Final *R* indexes [*I* ≧ 2*σ*(*I*)]	*R*_1_ = 0.0404, w*R*_2_ = 0.0984	*R*_1_ = 0.0458, w*R*_2_ = 0.1219
Largest diff. peak/hole/eÅ^−3^	0.28/−0.39	0.28/−0.16
Final *R* indexes [all data]	*R*_1_ = 0.0558, w*R*_2_ = 0.1102	*R*_1_ = 0.0851, w*R*_2_ = 0.1426
Flack parameter	—	0.07(12)

**Table 2:** Hydrogen bonds in the prepared compounds

D	H	A	*d*(D–H)/Å		*d*(H–A)/Å	*d*(D–A)/Å	D–H–A/°
**BDMBSH (** **4** **)**							
C5	H5	O2^a^	0.93		2.57	3.282(2)	133.2
N1	H1N	O1^b^	0.80(2)		2.21(2)	3.000(2)	168.8(19)

**BDMMBSH (** **5** **)**							
C14	H14B	O1^c^		0.97	2.60	3.273(9)	126.3

a 1 − *X*, 1/2 + *Y*, 1/2 − *Z*.

b 1 − *X*, 1 − *Y*, 1 − *Z*.

c 1 + *X*, *Y*, *Z*.

## 4. Application

### 4.1 Detection of Pb^2+^ using BDMMBSH by electrochemical method

**Scheme 2:**
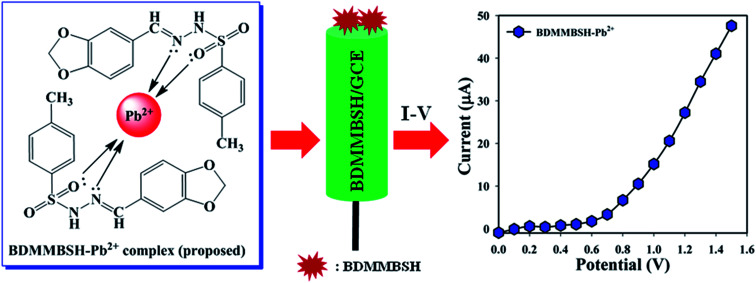
Proposed mechanism of BDMMBSH–Pb^2+^ complex formation and the respective electrochemical responses.

The development of a custom-made electrode with small organic molecules is the initial application as a TMI sensor. Thus, the BDMMBSH-modified GCE was examined in PB for the sensitive detection of the TMI, Pb^2+^. The as-synthesized two ligands worked as conjugate molecules to detect Pb^2+^, where their nitrogen and oxygen atoms donate electrons to Pb^2+^ because they possess lone pairs of electrons (Scheme 2). Here, two molecules were synthesized to observe the electrochemical responses toward heavy metal ions. Based on the electrochemical method, the current signals (CS) of the BDMMBSH/GCE/NF sensor extensively changed throughout the adsorption of Pb^2+^. Accordingly, the proposed mechanism for the electrochemical detection of Pb^2+^ using the *I*–*V* performance based on the BDMMBSH–Pb^2+^ complex arrangement is presented in Scheme 2.

**Fig. 3:**
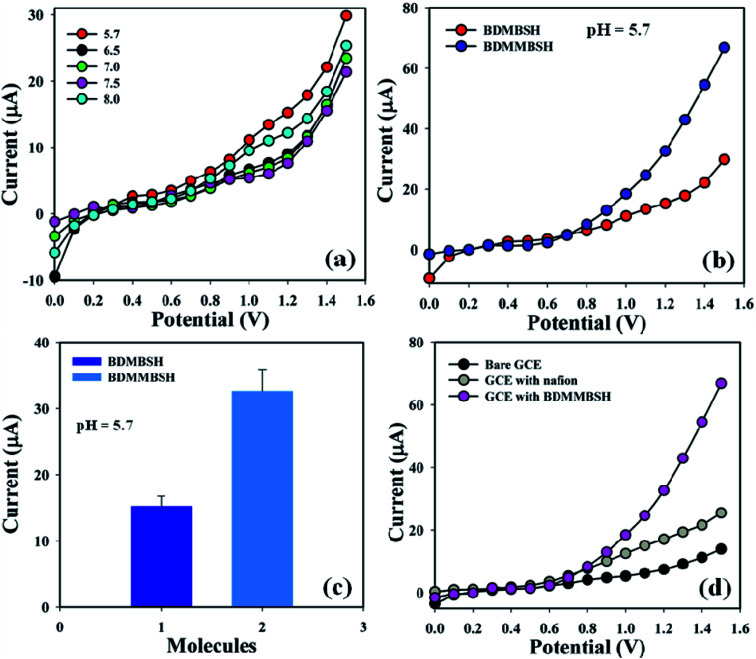
Optimization of the sensor: (a) pH examination, (b) electrochemical response of BDMBSH and BDMMBSH compounds, (c) bar diagram presentation of BDMBSH and BDMMBSH molecule optimization at +1.2 V with error bars of 10.0%, and (d) comparison of the electrochemical response with the bare and coated electrodes.

Initially, the effect of the phosphate buffer pH (10.0 mL) was examined with respect to BDMBSH to determine the most appropriate conditions to detect the toxic metal ion, and pH = 5.7 showed the highest response towards the tailored electrode compared with other PB pH (Fig. 3a). Accordingly, BDMBSH and BDMMBSH were optimized in PB (pH = 5.7 and 10.0 mL), and BDMMBSH showed higher responses (Fig. 3b), where the bar diagram of the molecule optimization at +1.2 V with error bars of 10.0% is presented in Fig. 3c. The *I*–*V* responses for the uncoated GCE, GCE with Nafion, and GCE with Nafion coated with BDMMBSH working electrodes are presented in Fig. 3d, where differences in the current signal among the tailored electrodes can be observed, and the signal was the highest with the BDMMBSH/GCE/NF sensor compared with the bare GCE and GCE with Nafion.

**Fig. 4:**
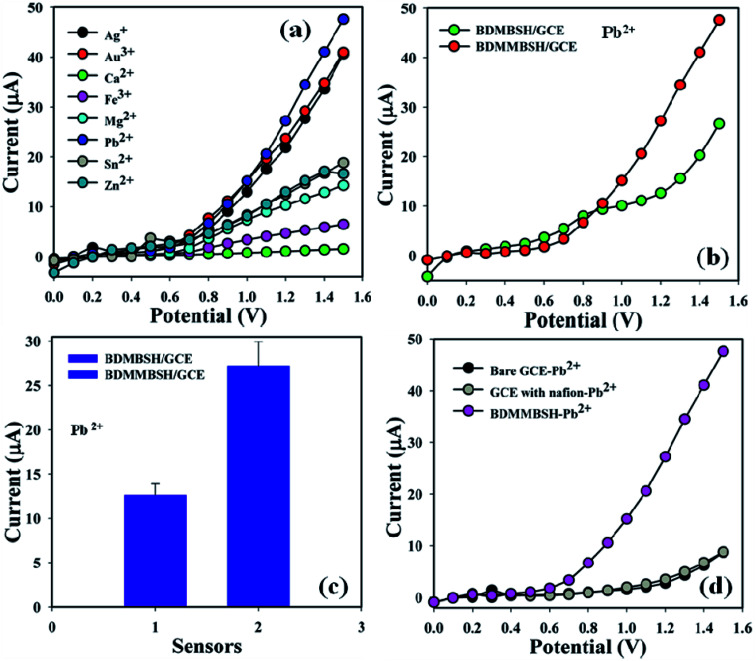
Sensor performances. (a) Selectivity investigation, (b) control experiment with the two synthesized compounds, (c) bar diagram presentation of the control experiment at +1.2 V with an error limit of 10.0%, and (d) control experiment in the presence of lead ions with the bare and coated electrodes.

Toxic metal ions (25.0 μL and 1.0 μM) including Ag^+^, Au^3+^, Ca^2+^, Fe^3+^, Mg^2+^, Pb^2+^, Sn^2+^, and Zn^2+^ were examined in phosphate buffer (10.0 mL, pH = 5.7, and 100.0 mM) using the unique active surface area containing different custom-made electrodes in order to determine the maximum current signal towards the BDMMBSH/GCE/NF sensor, and accordingly it was distinctly found that the sensor was more selective towards Pb^2+^ compared to other toxic metal ions (Fig. 4a). A control experiment was performed at 1.0 μM (25.0 mL) in phosphate buffer (10.0 mL, pH = 5.7, and 100.0 mM) using different deposited electrodes to exhibit that the BDMMBSH derivatives and BDMMBSH showed excellent responses towards Pb^2+^ (Fig. 4b). Fig. 4c shows the bar diagram for the control experiment at +1.2 V with error bars of 10.0%. The current signals without Pb^2+^ (black) and with Pb^2+^ (grey and pink) were also examined (Fig. 4d). An increase in the current signal was observed for the BDMMBSH/GCE/NF sensor in the presence of Pb^2+^, which possesses a massive external area, resulting in enhanced exposure for the potential incorporation and adsorption of the favored toxic metal ions in the holey BDMMBSH molecule surfaces.

**Fig. 5:**
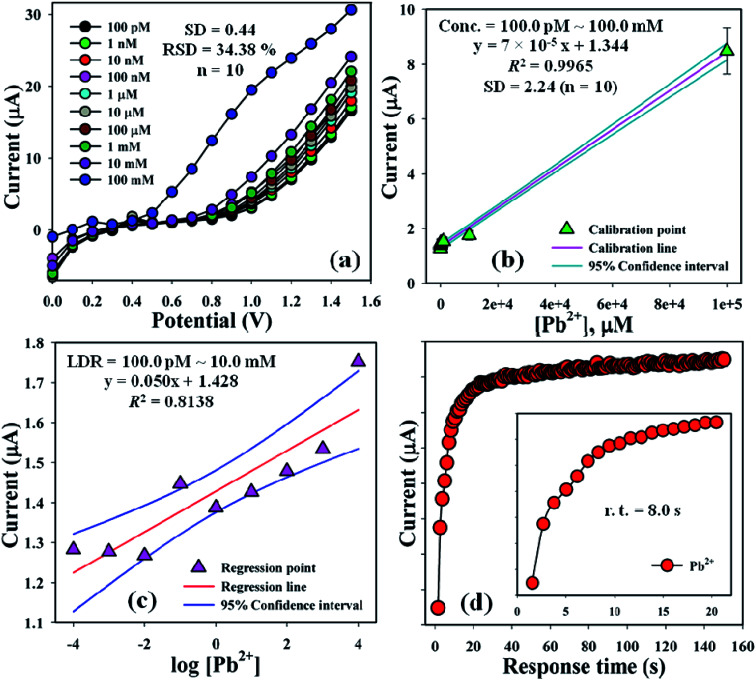
Sensor optimization. (a) Concentration variation study, (b) calibration curve at +0.7 V with error bar = 10.0%, (c) linear dynamic range plot with confidence interval, and (d) response time of Pb^2+^ towards the BDMMBSH/GCE/NF sensor.

The current signals of the TMI, Pb^2+^ with different concentrations (100.0 pM–100.0 mM) towards the BDMMBSH/GCE/NF sensor were examined to determine the variation in the current signals of the modified electrode for the function of Pb^2+^ detection under the standard conditions. It was observed that the current signals increased at a regular basis from a lower to higher conc. of Pb^2+^ [SD = 0.42, RSD = 26.75% at +0.4 V, *n* = 10, and EB = 10.0%] (Fig. 5a). A good range of Pb^2+^ conc. was evaluated from a lower to higher potential (0.0–+1.5 V) serially to determine the probable analytical upper limits. The calibration curve was plotted at +0.7 V as a function of Pb^2+^ conc. (100.0 pM–100.0 mM), which was found to be linear [*R*^2^ = 0.9965 and SD = 2.24 at *n* = 10] (Fig. 5b). The sensitivity, LOQ, and LOD were calculated from the calibration curvature and found to be 2220.0 pA μM^−1^ cm^−2^, 320.0 mM, and 96.0 pM, respectively.^24,25^ The LDR (100.0 pM–10.0 mM) was calculated from the calibration curve and was also found to be linear, *R*^2^ = 0.8133 (Fig. 5c). The response time of Pb^2+^ towards the BDMMBSH/GCE/NF sensor was measured at (1.0 μM and 25.0 μL) in buffer (10.0 mL, pH = 7.5, and 100.0 mM), which was determined to be 8.0 s (Fig. 5d).

### 4.2 Evaluation of sensor efficiency

**Fig. 6:**
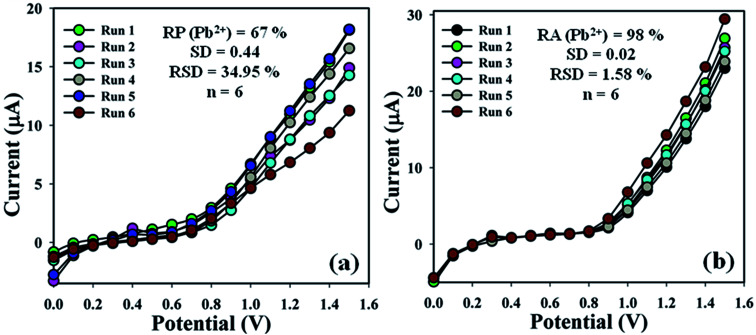
Sensor performance evaluation. (a) Reproducibility and (b) repeatability.

The sensing efficiency of the BDMMBSH/GCE/NF sensor was examined up to a few days to estimate its reproducibility (RP). Accordingly, a series of six consecutive cycles for the detection of Pb^2+^ at (1.0 μM and ∼25.0 μL) using different sensors in the same environment in buffer (10.0 mL, pH = 7.5, and 100.0 mM) was performed, and the BDMMBSH/GCE/NF sensor yielded good responses (RP = 67.0%, SD = 0.44 at +0.7 V, and *n* = 6) (Fig. 6a and Table S3†). The current signal of the BDMMBSH/GCE/NF sensor was measured with respect to storage time to examine its extended storage ability. Accordingly, the storage ability of the BDMMBSH/GCE/NF sensor was examined (1.0 μM and 25.0 μL) in buffer (10.0 mL, pH = 7.5, and 100.0 mM) under the standard conditions using the same sensor, and the repeatability (RA) at the calibrated potential (+0.7 V) was found to be 98.0% towards Pb^2+^, [SD = 0.02, RSD = 1.58%, and *n* = 6] (Fig. 6b and Table S3†). The sensitivity remained almost similar as the initial responses up to a few days, and after that the responses of the BDMMBSH/GCE/NF sensor declined gradually. This distinctly demonstrated that the proposed sensor can be used without any primary changes in its sensitivity for up to few days. A comparison of the Pb^2+^ detection using different organic compound-based sensors^26–33^ is discussed and presented in Table 3. According to the comparative study of previous sensors with various organic materials, the highest sensitivity value was found with our fabricated sensor probe (BDMMBSH/GCE/NF) for the detection of lead ions. Various inorganic materials were also used for the selective detection of lead ions *via* optical and electrochemical methods.^34–42^

**Table 3:** Detection of Pb^2+^ using different compound-modified sensors *via* electrochemical methods^a^

Sensor	Method	LDR (μg L^−1^)	Sensitivity (Ma L μg^−1^)	LOD (μg L^−1^)	Ref.
SPTE	CV	1–200	0.087	0.3	26
Graphene/PATE	—	1–300	0.035	0.1	27
Bi/C composite SPE	—	1–50	0.025	2.3	28
Bi/PSS/SPCE	—	∼45	0.39	0.27	29
Paper/SPCE	—	10–100	0.009	2.0	30
CMTE	—	5–110	0.47	0.87	31
Bi citrate/SPE	CV	10–80	0.040	0.9	32
BDMMBSH/GCE/NF	*I*–*V*	100–10 (pM–mM)	2220.0 (pA μM^−1^ cm^−2^)	96.0 pM	This work

a SPTE: stencil-printed transparency electrode, PATE: polyaniline transparency electrode, SPE: screen-printed electrode, SPCE: screen-printed carbon electrode, CMTE: carbon micro-thread electrode, and *I*–*V*: current–voltage.

### 4.3 Assessment of interference effect

**Fig. 7:**
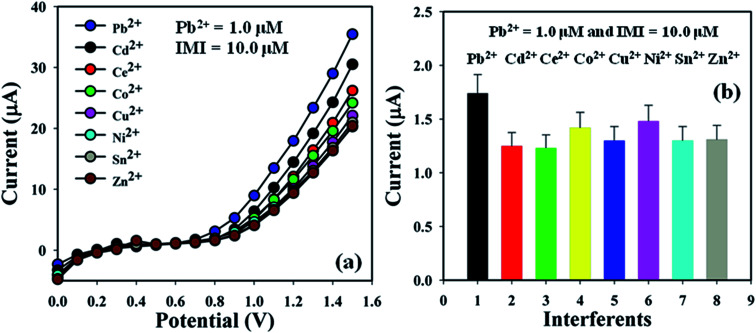
Sensor optimization. (a) Interference effect study and (b) bar diagram of the interference effect study at +0.7 V with error bars of 10.0%.

Investigation of the interference effects is one of the extensive practices in analytical science to determine the effect of different interfering metal ions having a similar cationic nature. Cd^2+^, Ce^2+^, Co^2+^, Cu^2+^, Ni^2+^, Sn^2+^, and Zn^2+^ are generally used as interfering metal ions in the detection of Pb^2+^ electrochemically. The current signals of the BDMMBSH/GCE/NF sensor upon the addition of Pb^2+^ (1.0 μM and 25.0 μL) and interfering metal ions such as Co^2+^, Cu^2+^, Ni^2+^, Sn^2+^, and Zn^2+^ (10.0 μM and 25.0 μL) in buffer (10.0 mL, pH = 7.5, and 100.0 mM) were examined using the same sensor. The interference effect of the interfering metal ions towards Pb^2+^ was calculated at the calibrated potential (+0.7 V), where the interfering metal ion of Pb^2+^ was considered to be 100.0% (Fig. 7a and b and Table 4). It was evident that BDMMBSH/GCE/NF sensor did not demonstrate any notable responses towards the IMI. Thus, the proposed sensor (BDMMBSH/GCE/NF) is appropriate for the detection of Pb^2+^ with good sensitivity.

**Table 4:** Interference effect study of interference metal ions towards the proposed Pb^2+^ sensor^a^

IMI	Observed current (μA)				IEF (%)	SD (*n* = 3)	RSD% (*n* = 3)
	*R*1	*R*2	*R*3	Average			
Pb^2+^	3.06	1.07	1.10	1.74	100	1.14	65.41
Cd^2+^	1.35	1.18	1.22	1.25	72	0.09	7.11
Ce^2+^	1.22	1.20	1.26	1.23	71	0.03	2.49
Co^2+^	1.67	1.27	1.33	1.42	82	0.22	15.16
Cu^2+^	1.29	1.28	1.33	1.30	75	0.03	2.04
Ni^2+^	1.73	1.36	1.34	1.48	85	0.22	14.87
Sn^2+^	1.27	1.31	1.34	1.30	75	0.04	2.69
Zn^2+^	1.27	1.31	1.34	1.31	75	0.04	2.69

a IEF of Pb^2+^ was considered to be 100.0%, IMI: interfering metal ions, *R*: reading, IEF: interference effect, SD: standard deviation, and RSD: relative standard deviation.

### 4.4 Analysis of real environmental samples

**Fig. 8:**
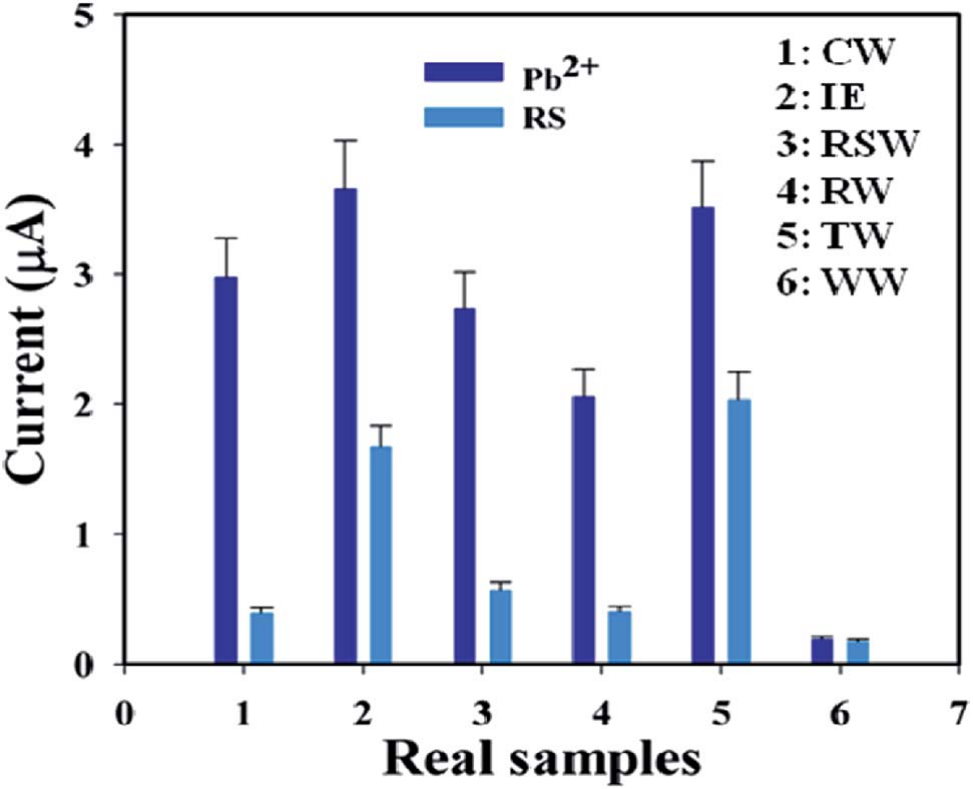
Validation of real samples with the BDMMBSH/GCE/NF sensor probe *via* the electrochemical (recovery) method.

A group of natural samples such as coal water, industrial effluent, Red Sea water, rice water, tap water, and well water was utilized as real samples to estimate the *I*–*V* performance of the BDMMBSH/GCE/NF sensor. A standard addition procedure was used to determine the concentration of Pb^2+^. A fixed quantity (∼25.0 μL) of each natural sample was examined in phosphate buffer (10.0 mL, pH = 7.5, and 100.0 mM) using the modified BDMMBSH/GCE/NF sensor.^43–49^ The concentrations were calculated at the calibrated potential (+0.7 V) for the detection of Pb^2+^ in coal water, industrial effluent, Red Sea water, rice water, tap water, and well water, which absolutely demonstrated that the *I*–*V* method is proper, reliable, and appropriate for the analysis of real samples. Fig. 8 shows the bar diagram presentation of the natural sample analysis at +0.7 V with error bars of 10.0%. The results show that the conc. of Pb^2+^ in the well water was the greatest among the natural samples (Table 5).

**Table 5:** Performance of the sensor with natural samples *via* the electrochemical (recovery) method^a^

ME	AC, Pb^2+^ (25.0 μL, μM)	OC, Pb^2+^ (μA)	RSA (25.0 μL)	ROC (RSA, μA)				FC (μM)	*R* (%)	SD (*n* = 3)	RSD (%) *n* = 3
				*R*1	*R*2	*R*3	*A*				
1	1.0	2.98	CW	0.41	0.39	0.39	0.39	0.13	13	0.01	2.91
2	1.0	3.66	IE	1.70	1.64	1.67	1.67	0.46	46	0.03	1.80
3	1.0	3.50	RSW	1.98	2.02	2.05	2.02	0.60	60	0.02	1.59
4	1.0	2.96	RW	0.42	0.39	0.38	0.39	0.14	14	0.02	2.79
5	1.0	3.52	TW	2.02	2.03	2.07	2.04	0.58	58	0.03	1.30
6	1.0	2.49	WW	1.71	1.68	1.65	1.68	0.44	44	0.03	2.23

a ME: modified electrode, AC: added concentration, OC: observed current, RSA: real sample added, CW: coal water, IE: industrial effluent, RSW: Red sea water, RW; rice water, TW: tap water, WW: well water, *R*1–*R*3: reading, *A*: average, *R*: recovery, ROC: respective observed current, FC: found concentration, SD: standard deviation, and RSD: relative standard deviation.

## 5. Conclusion

Herein, two new BDMMBSH compounds were synthesized, characterized, and used to selectively detect heavy metal ions *via* an electrochemical method. Good sensor performances for the selective Pb^2+^ sensor were demonstrated in terms of sensitivity, LOQ, LOD, LDR, response time, RP, and RA. This new device can be a suitable analytical tool to design sensitive and selective TMI detection platforms with BDMMBSH-embedded GCE using conducting polymer matrix. Finally, the innovative sensor probe was introduced from this new approach for the detection of toxic TMIs in the environmental and healthcare fields on a broad scale.

## Conflicts of interest

The authors declared that there is no conflict of interest to be reported in this research work.

## Supplementary Material

RA-010-C9RA09080K-s001

RA-010-C9RA09080K-s002
