# Supplementary material for: The synthesis and application of (E)-N′-(benzo[d]dioxol-5-ylmethylene)-4-methyl-benzenesulfonohydrazide for the detection of carcinogenic lead
Source: RSC Adv. 2020 Feb 3;10(9):5316–27. doi: 10.1039/c9ra09080k (PMC9049008; doi:10.1039/c9ra09080k)
Supplement: RA-010-C9RA09080K-s001 [file RA-010-C9RA09080K-s001.pdf]

Electronic Supplementary Material (ESI) for RSC Advances.
This journal is © The Royal Society of Chemistry 2019

Synthesis and application of (*E*)-*N′*-(benzo[d]dioxol-5-ylmethylene)-4-methylbenzenesulfonohydrazide for carcinogenic lead detection

Electronic supplementary materials (ESM)

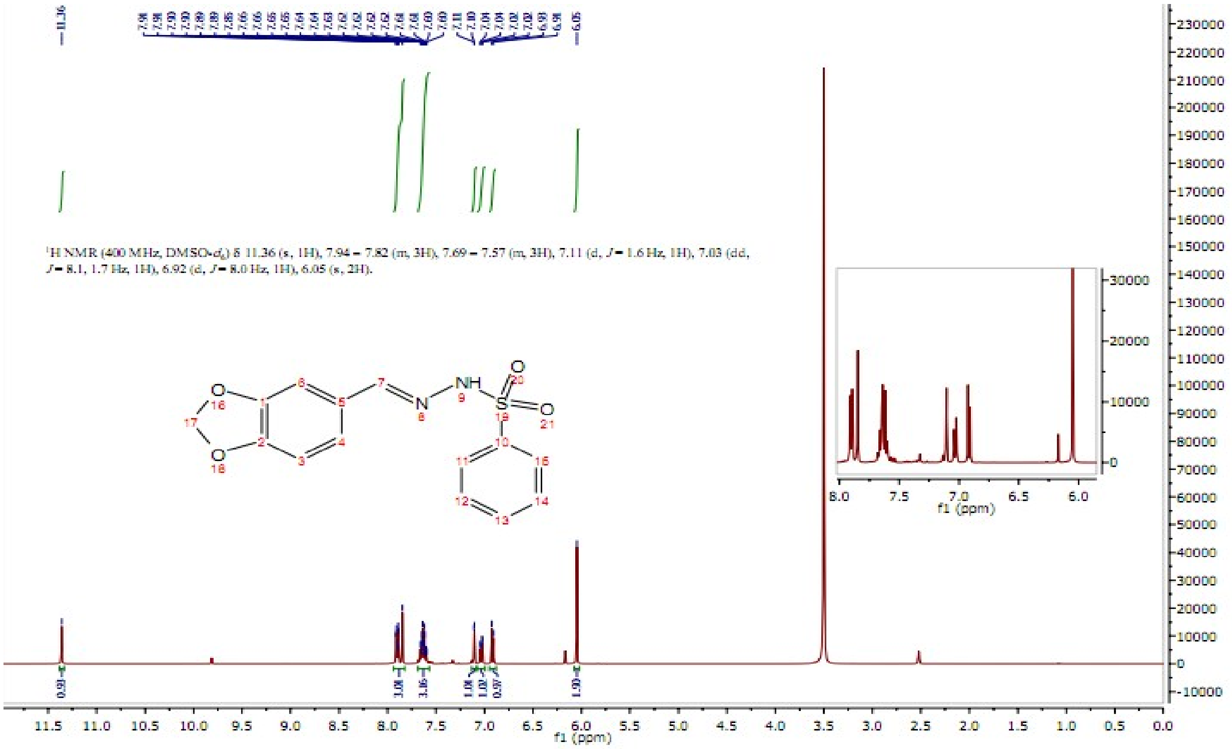

Fig. S1 ^{1}H-NMR of BDMBSH (4)

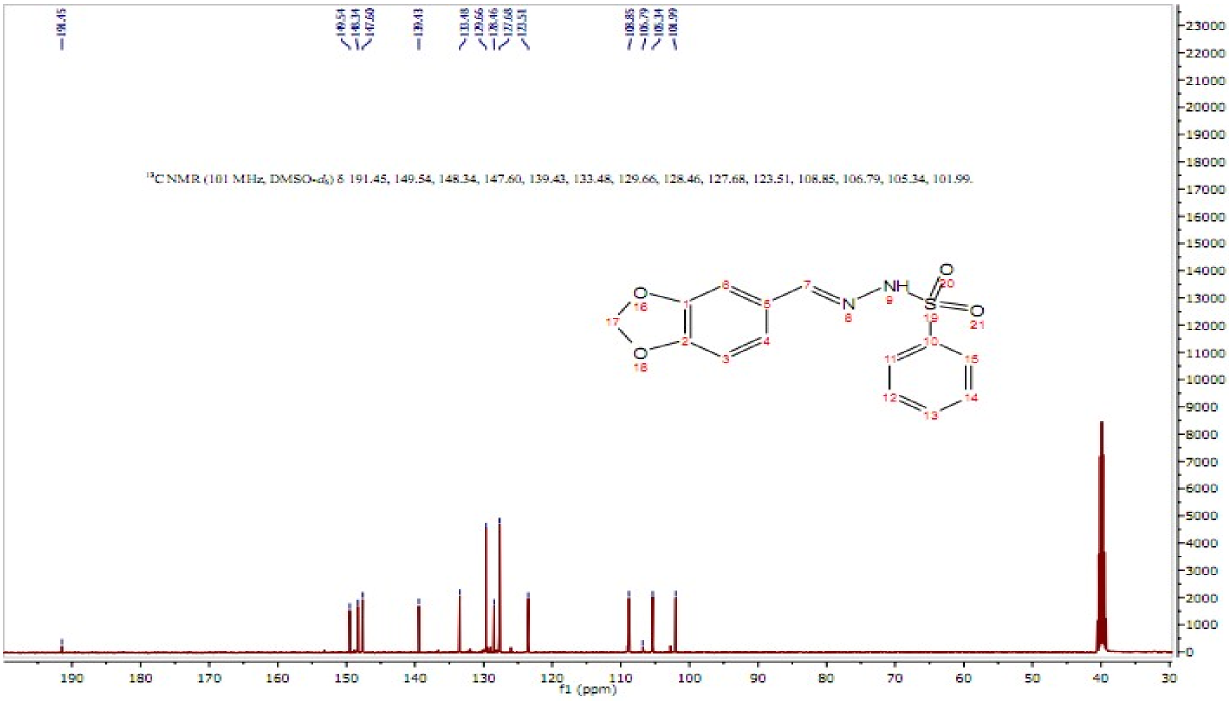

Fig. S2 ^{13}C-NMR of BDMBSH (4)

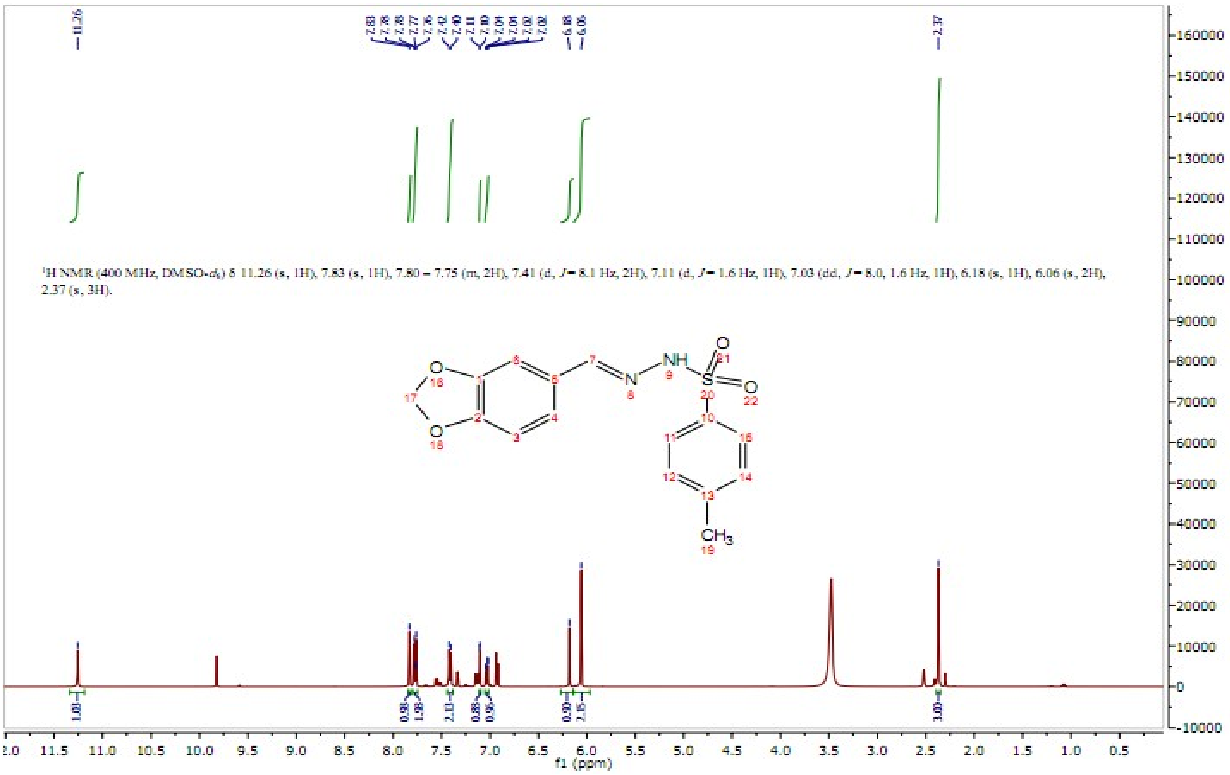

Fig. S3 ^{1}H-NMR of BDMMBSH (5)

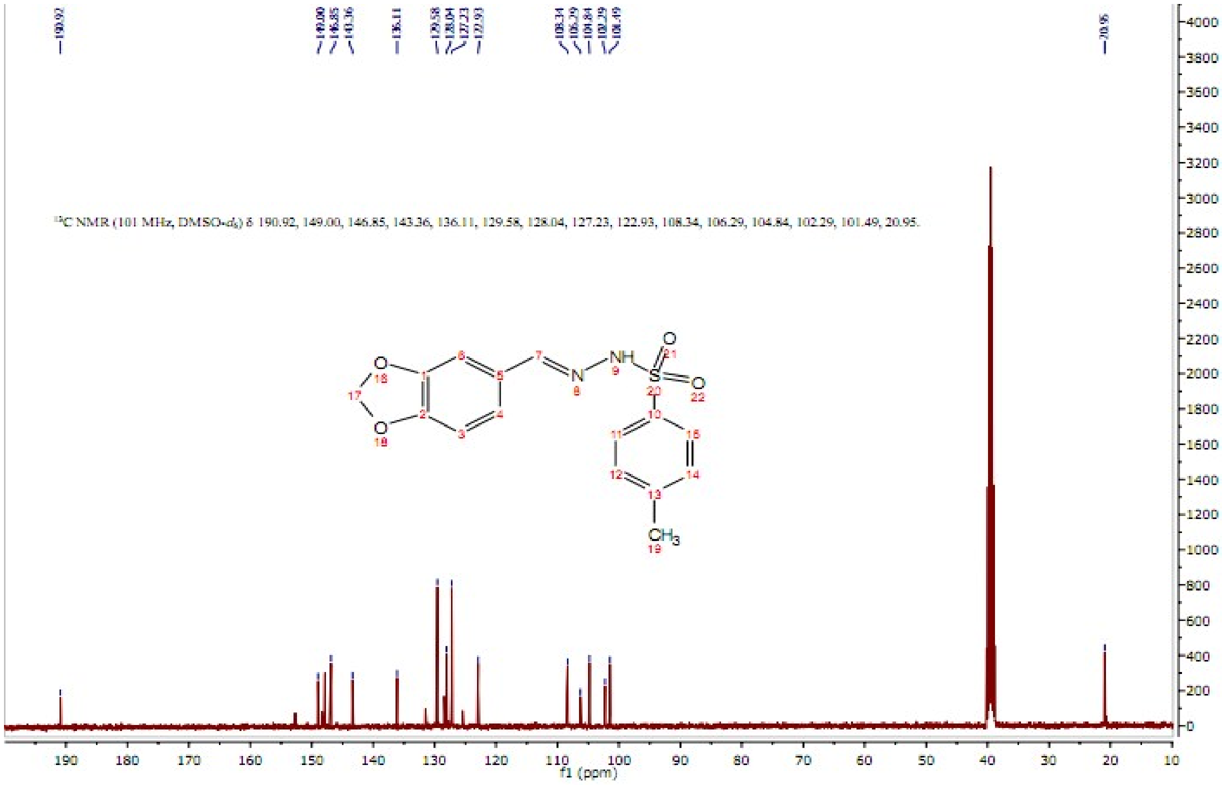

Fig. S4 ^{13}C-NMR of BDMMBSH (5)

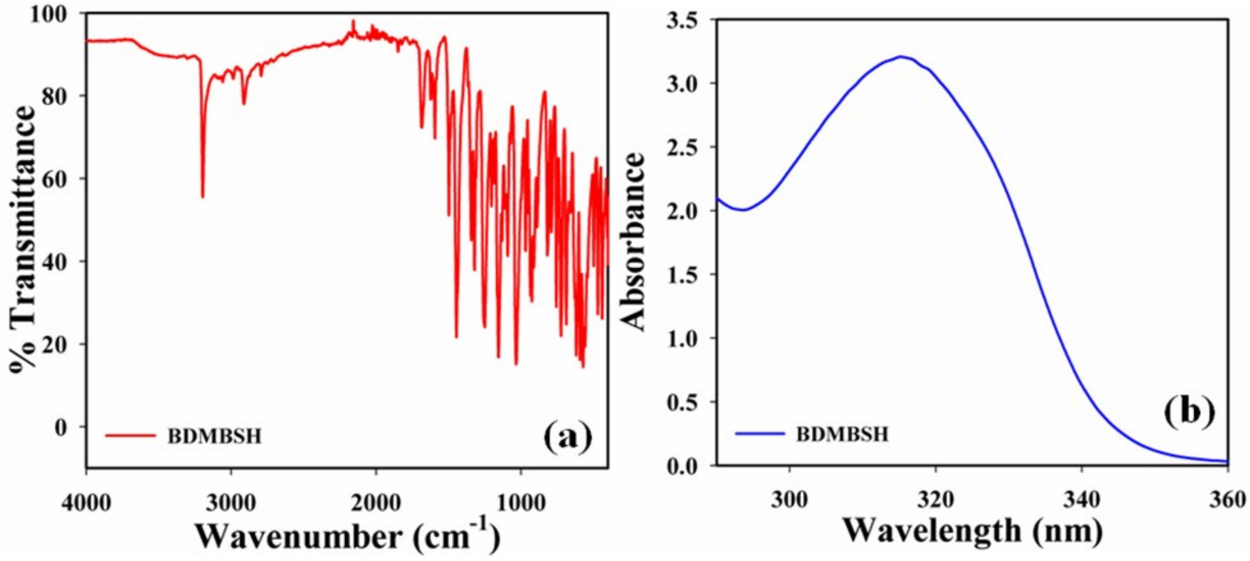

Fig. S5 FTIR and UV-Vis spectra of BDMBSH (4)

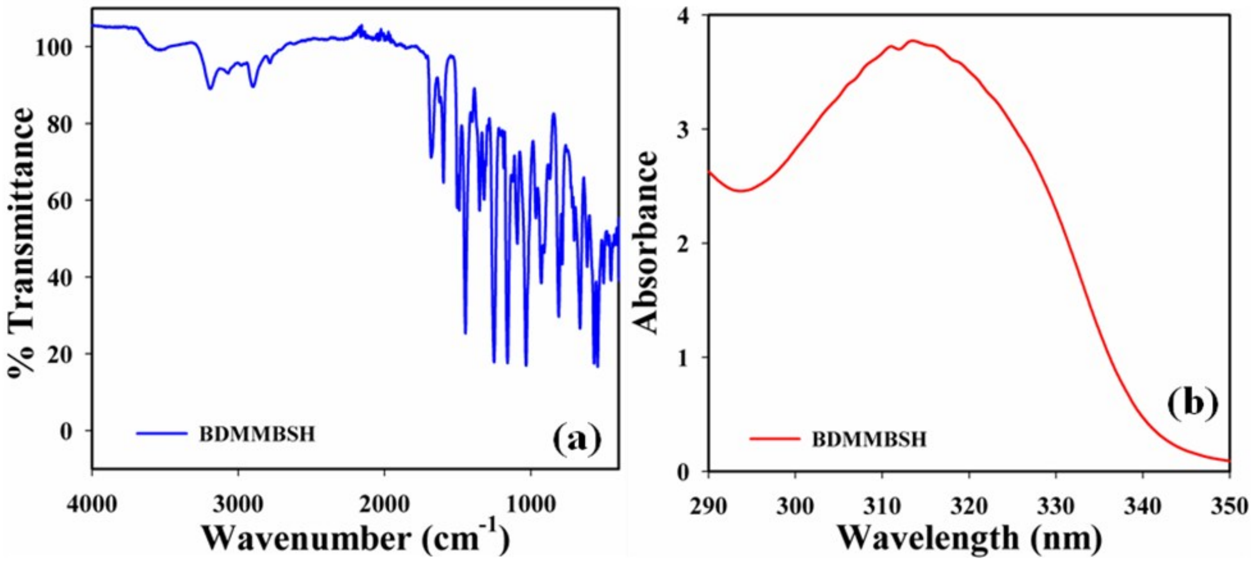

Fig. S6 FTIR and UV-Vis spectra of BDMMBSH (5)

Table S1 Bond lengths for the synthesized molecules

Atom	Atom	Length/Å	Atom	Atom	Length/Å
		**BDMBSH (4)**			
C1	C2	1.382(2)	C10	C11	1.388(3)
C1	C6	1.388(2)	C10	O3	1.373(2)
C1	S1	1.7633(17)	C11	C12	1.362(3)
C2	C3	1.381(3)	C11	O4	1.368(2)
C3	C4	1.372(3)	C12	C13	1.397(3)
C4	C5	1.379(3)	C14	O3	1.421(3)
C5	C6	1.377(3)	C14	O4	1.424(3)
C7	C8	1.460(3)	N1	N2	1.408(2)
C7	N2	1.275(2)	N1	S1	1.6316(16)
C8	C9	1.406(2)	O1	S1	1.4389(13)
C8	C13	1.387(3)	O2	S1	1.4217(14)
C9	C10	1.362(3)			
		**BDMMBSH (5)**			
C1	C2	1.382(6)	C9	C10	1.379(7)
C1	C6	1.376(6)	C10	C11	1.372(8)
C1	S1	1.753(4)	C10	O3	1.351(7)
C2	C3	1.374(7)	C11	C12	1.311(8)
C3	C4	1.380(7)	C11	O4	1.414(7)
C4	C5	1.377(7)	C12	C13	1.385(7)
C4	C15	1.508(8)	C14	O3	1.466(8)
C5	C6	1.367(7)	C14	O4	1.363(10)

C7	C8	1.443(6)	N1	N2	1.404(4)
C7	N2	1.276(5)	N1	S1	1.626(3)
C8	C9	1.376(7)	O1	S1	1.424(3)
C8	C13	1.389(6)	O2	S1	1.430(3)

Table S2 Bond angles of the prepared compounds

Atom	Atom	Atom	Angle/°	Atom	Atom	Atom	Angle/°
			**BDMBSH (4)**				
C2	C1	C6	120.98(16)	C12	C11	C10	121.77(18)
C2	C1	S1	119.67(14)	C12	C11	O4	128.44(19)
C6	C1	S1	119.34(13)	O4	C11	C10	109.79(17)
C3	C2	C1	118.98(18)	C11	C12	C13	116.44(19)
C4	C3	C2	120.42(18)	C8	C13	C12	122.30(19)
C3	C4	C5	120.30(18)	O3	C14	O4	108.41(16)
C6	C5	C4	120.28(19)	N2	N1	S1	115.41(12)
C5	C6	C1	119.03(17)	C7	N2	N1	114.88(16)
N2	C7	C8	121.84(17)	C10	O3	C14	106.00(16)
C9	C8	C7	121.58(17)	C11	O4	C14	105.93(16)
C13	C8	C7	118.30(17)	N1	S1	C1	107.74(8)
C13	C8	C9	120.10(18)	O1	S1	C1	108.70(8)
C10	C9	C8	116.80(17)	O1	S1	N1	103.54(8)
C9	C10	C11	122.58(17)	O2	S1	C1	108.35(8)
C9	C10	O3	127.99(18)	O2	S1	N1	108.90(9)
O3	C10	C11	109.42(17)	O2	S1	O1	119.07(8)
			**BDMMBSH (5)**				
C2	C1	S1	119.4(3)	O3	C10	C11	111.8(5)
C6	C1	C2	120.2(4)	C10	C11	O4	107.8(6)
C6	C1	S1	120.4(3)	C12	C11	C10	123.8(5)
C3	C2	C1	119.0(4)	C12	C11	O4	128.3(5)
C2	C3	C4	121.4(5)	C11	C12	C13	116.1(5)
C3	C4	C15	120.1(5)	C12	C13	C8	122.1(6)
C5	C4	C3	118.3(5)	O4	C14	O3	109.4(6)
C5	C4	C15	121.6(5)	N2	N1	S1	114.7(3)
C6	C5	C4	121.2(4)	C7	N2	N1	114.2(3)
C5	C6	C1	119.8(5)	C10	O3	C14	103.9(6)
N2	C7	C8	122.9(4)	C14	O4	C11	106.3(5)
C9	C8	C7	120.8(4)	N1	S1	C1	108.47(18)
C9	C8	C13	120.4(4)	O1	S1	C1	109.00(18)
C13	C8	C7	118.9(4)	O1	S1	N1	104.69(18)
C8	C9	C10	116.1(5)	O1	S1	O2	119.41(19)

C11	C10	C9	121.4(5)	O2	S1	C1	107.7(2)
O3	C10	C9	126.8(6)	O2	S1	N1	107.11(18)

Table S3 Reproducibility and repeatability study of NMBSH/GCE sensor at CP (+ 0.7 V).

**Replicates**	**Current (μA)**	**Reproducibility, RP (%)**		**Current (μA)**	**Repeatability, RA (%)**	
		**Individual**	**Average**		**Individual**	**Average**
1	1.96	100		1.36	100	
2	1.48	76		1.34	99	
3	0.85	43	67	1.35	99	98
4	0.87	44		1.30	96	
5	1.61	82		1.33	98	
6	1.05	54		1.33	98	

Here, the reproducibility and repeatability of replicate 1 has been considered to be 100%.
